# Note on the PCR threshold standard curve

**DOI:** 10.1186/s13104-017-3036-4

**Published:** 2017-12-11

**Authors:** Benedict G. Archer

**Affiliations:** Vallejo, CA USA

**Keywords:** PCR, qPCR, Standard curve, Threshold, Efficiency

## Abstract

**Objective:**

The PCR threshold standard curve is based on an exponential model of the initial phase of a PCR run where template replication efficiency is constant cycle to cycle. As such it requires that a threshold is at a level of amplified template not higher than where replication efficiency falls from its initial value. A second requirement is that all amplification profiles, both calibration and test, have the same initial efficiency. However, whether these requirements are met may not be checked, and there seems an apparent awareness that thresholds can be set higher than where efficiency has dropped from the initial value without compromising result validity. The objective of this study is to reconcile using the method without satisfying the requirement that amplification is exponential at threshold level.

**Results:**

Substituting the more general requirement that profile shapes be congruent to threshold level, except for translation along the cycle axis, and a derivation of the standard curve that includes cycles beyond the exponential phase accomplishes the objective without affecting usage of the method or any prior results and enables a practicable way to verify that the second requirement for same initial efficiency is satisfied.

**Electronic supplementary material:**

The online version of this article (10.1186/s13104-017-3036-4) contains supplementary material, which is available to authorized users.

## Introduction

The threshold standard curve method, regarded a gold standard for quantitative PCR, is based on an exponential model of the initial phase of a PCR run. The requirement that the threshold is at a level where amplification is exponential, that is, in the initial, constant efficiency phase of a run, is explicit in derivations of the straight line model of the threshold standard curve presented in articles, tutorials, commercial guides, etc. Many sources, e.g. [1], include the requirement in discussing the method, but some also downplay the importance of where a threshold level is set. There seems a tacit understanding, perhaps engendered by the great number of valid analyses where the requirement is not verified, or is even recognized to not be met, e.g. [2], that strict conformance is not critical. However, an explanation why calibrations and analyses using a threshold set at a level higher than where the exponential phase of the run has ended can be valid and a derivation of the model for the standard curve that avoids the requirement are lacking.

We show in this note that the validity of a standard curve, predicted target amounts in unknown samples based on it, the efficiency estimated from its slope and the linear relationship between $C_T$ and the logarithm of target amount in a calibration sample do not depend on threshold level provided a more general requirement is met. We make a logical case for replacing the requirement regarding efficiency with a less restrictive requirement, and we derive the line modeling the standard curve in a way that includes cycles beyond the exponential (constant efficiency) phase of a run. Our model of the standard curve remains a straight line representation of $C_T$ as a function of the logarithm of calibration target amount and does not change how a calibration is constructed or used to estimate unknown target amounts.

## Main text

### Analysis

Our analysis identifies a characteristic of amplification profiles implied by the requirement of constant efficiency but more general, substitutes it for requiring that thresholds are within the exponential phase, and extends the derivation of the threshold standard curve to incorporate the new requirement.

#### Requiring shape congruency instead of constant efficiency to threshold

Amplification profiles having the same initial efficiency and maintaining that efficiency to a threshold level will have the same shape to that threshold except for translation along the cycle axis. Two profiles with different initial efficiencies cannot be superimposed by shifting along the cycle axis. If a threshold is set higher than is reached within the exponential phase but at a level where the profiles have the same shape, differences in $C_T$ from one profile to the next (and slopes of standard curves) will be the same as for any lower threshold. Thus substituting shape congruency to a threshold for the requirement that thresholds be set within the exponential phase also allows setting thresholds beyond the exponential phase.

#### Modifying the straight-line model of the standard curve

We briefly recapitulate the derivation of the equation for the standard curve in order to show how to incorporate the new requirement that extends the model to include cycles beyond the exponential phase.

#### Derivation of the standard curve for threshold within exponential phase

 Denoting efficiency at cycle *k* by $E_k$, amount of starting target DNA by $X_0$, and a threshold amount of amplicon $X_T$ corresponding to a number of cycles $C_T$, any PCR run is represented by the general equation,

1 $$\begin{aligned} X_T = X_0 \prod _{k=1}^{C_T} (1+E_k). \end{aligned}$$

This equation requires no assumptions; it represents a growth process in which the amount of amplicon increases by the proportional amount $E_k$ at each cycle. Adding the usual assumption that the threshold is set within the exponential cycle range where efficiency is at the initial value ($E_1$) and constant, $E_k = E_1$ for all cycles up to $C_T$, (1) becomes the usual starting point for deriving the equation for the standard curve in PCR guides and publications,

2 $$\begin{aligned} X_T = X_0 (1+E_1)^{C_T}. \end{aligned}$$

Log-transformation and re-arrangement of (2) gives the standard curve,

3 $$\begin{aligned} C_T = \frac{\log (X_T)}{\;\;\;\log (1+E_1)}\; -\; \frac{1}{\log (1+E_1)}\,\log (X_0), \end{aligned}$$

a straight line with intercept representing the logarithm of the threshold amount of amplified target divided by the logarithm of $1+E_1$, and slope equal to minus the reciprocal of the logarithm of $1+E_1$. $C_T$ is a value interpolated between the cycles bounding the threshold crossing. The slope and intercept in (3) are determined by fitting a line to a set of $C_T$ values measured for samples with known $X_0$. With the slope and intercept determined, the target amount $X_0$ in an unknown sample is computed from a measured $C_T$ and the slope and intercept.

#### Derivation of the standard curve for threshold after exponential phase

  The revised model which includes cycles beyond the constant efficiency range continues to imagine a threshold set corresponding to a point in the cycle sequence at or before the end of the constant efficiency phase. This threshold may be too low to observe and is not set explicitly. However, we regard the crossing of this threshold at cycle $C_{T_0}$ to occur at an interpolated value just as a usual threshold crossing is a value interpolated between bounding cycles. Measured $C_T$ values are determined in the usual way from a working threshold set without concern that it is within the exponential range. Requiring that the threshold be set at a level where all profiles to be analyzed have the same shape implies that in cycles following $C_{T_0}$ efficiency decreases each cycle are the same in all profiles. It follows that the cycle distance from $C_{T_0}$ to $C_T$ is a whole number and the same for all profiles. We denote this difference by $\Delta =C_T - C_{T_0}$, and rewrite (2) to include these cycles,

4 $$\begin{aligned} X_T = X_0 (1+E_1)^{C_{T_0}} \prod _{i=C_{T_0}+1}^{C_{T_0} + \Delta }(1+E_i). \end{aligned}$$

Because the decrease in efficiency through these cycles is the same in every profile, we can replace the product term in (4) with the geometric mean of $(1+E_i)$ over cycles $C_{T_0}+1$ to $C_{T_0} + \Delta$ raised to the power $\Delta$. Denoting the geometric mean by $\left<1+E_\delta \right>$, we get,

5 $$\begin{aligned} X_T = X_0 (1+E_1)^{C_{T_0}} \left<1+E_\delta \right>^{\Delta }. \end{aligned}$$

The measured variable $C_T$ is re-introduced into (5) using $C_T = C_{T_0} + \Delta$,

6 $$\begin{aligned} X_T = X_0 (1+E_1)^{C_T}\left[ \frac{\left<1+E_\delta \right>}{(1+E_1)}\right] ^{\Delta }, \end{aligned}$$

which is log-transformed and re-arranged to get the new standard curve equation,

7 $$\begin{aligned} C_T = \frac{\log \left( X_T\left[ \frac{(1+E_1)}{\left<1+E_\delta \right>}\right] ^{\Delta }\right) }{\;\;\;\log (1+E_1)}\; -\; \frac{1}{\log (1+E_1)}\,\log (X_0). \end{aligned}$$

The only difference between this standard curve and the usual one (3) is in the intercept coefficient in which in (7) the amount of amplicon at threshold is multiplied by the factor $\left[ \frac{(1+E_1)}{\left<1+E_\delta \right>}\right] ^{\Delta }.$ Because this factor is $\ge 1$, $X_T$ estimated from the intercept of the usual model will be over-estimated if the threshold is set higher than reached within the constant efficiency part of the PCR run. This difference will not matter when the intercept value is used to compute the amount of target in a test sample as the value of the intercept, determined by the data, is the same whatever model is used; only what the intercept represents is different. The level where $\left[ \frac{(1+E_1)}{\left<1+E_\delta \right>}\right]$ becomes greater than one marks the end of the exponential phase and can be estimated by comparing $X_T$ calculated from intercepts of standard curves constructed from thresholds at various levels. The efficiency estimated from the slope is the same as in (3), and from both curve models is a first-cycle efficiency whatever the threshold level.

### Discussion

We demonstrate the extended model using data published by Rutledge and Stewart [2] who in a study of methods for estimating efficiency presented data that are an example of the premise of this analysis. We also briefly note other insights afforded by the revised equation for the standard curve line.

#### Applying the new standard curve to data

Rutledge and Stewart [2] published an example plot of PCR profiles generated from five quantities of lambda gDNA ranging from $1.88\times 10^1$ to $1.88\times 10^5\,$ genomes; we apply the revised model to their data to demonstrate its use and how it reveals information about the data not revealed by the usual analysis. Because we wanted to estimate standard curve parameters from threshold levels in addition to those examined by Rutledge and Stewart, we manually de-plotted $C_T$ values from their Figure 2A for the seven threshold levels listed in Table 1. Slopes, and efficiencies estimated from slopes of the standard curves constructed from the de-plotted $C_T$ values for each threshold level were essentially the same as displayed for five of the threshold levels in an inset to their Figure 2B in [2]. The second and third columns of Table 1 contain values of $E_1$ and $X_T\left[ \frac{\left( 1+E_1\right) }{\left<1+E_\delta \right>}\right] ^\Delta$ derived from the slopes and intercepts of the standard curves. Actual values of threshold target amounts, column 4 in Table 1, were estimated assuming linear instrument response and that amplification was within the constant efficiency part of the run at the lowest threshold, 500 fluorescence units (FU). Doubling the threshold to $1000\,$ FU also doubled the intercept confirming that the two lowest thresholds were below the amplicon level where efficiency begins to fall off, but on doubling the threshold again to $2000\,$ FU the intercept more than doubled. The ratio $\left[ \frac{\left( 1+E_1\right) }{\left<1+E_\delta \right>}\right]$ was now greater than one indicating that $E_\delta < E_1$ and amplification was beyond the exponential phase. The fifth column contains $\left[ \frac{\left( 1+E_1\right) }{\left<1+E_\delta \right>}\right] ^\Delta$ computed from the third and fourth columns. $\Delta$ in the next column was estimated visually from the plotted data, and $E_\delta$ in the rightmost column was computed from values in the second, fifth and sixth columns.

**Table 1:** Attributes of calibrations at various thresholds in Figure 2A of [2]

Threshold	$E_1$	$X_T\left[ \frac{\left( 1+E_1\right) }{\left<1+E_\delta \right>}\right] ^\Delta$	$X_T$	$\left[ \frac{\left( 1+E_1\right) }{\left<1+E_\delta \right>}\right] ^\Delta$	$\Delta$	$E_\delta$
500	0.926	1.75e+09	1.74e+09	1.00	0	−
1000	0.920	3.48e+09	3.49e+09	1.00	1	0.92
2000	0.925	7.67e+09	6.97e+09	1.11	2	0.83
3000	0.938	1.56e+10	1.05e+10	1.49	3	0.70
5000	0.930	3.22e+10	1.74e+10	1.85	4	0.65
7000	0.929	6.90e+10	2.44e+10	2.83	5	0.57
9000	0.911	1.68e+11	3.14e+10	5.35	7	0.50

The equivalence of $E_\delta$ with $E_1$ at the $1000\,$ FU threshold level and the decrease in $E_\delta$ at $2000\,$ FU tell us that the constant efficiency portion of the run ended at an amplicon amount corresponding to a signal level between $1000\,$ and $2000\,$ FU, consistent with the analysis in [2]. The progressive decrease in $E_\delta$ for higher thresholds shows the continuing decrease in efficiency after the $2000\,$ FU threshold is passed but still several cycles before the abrupt decrease in efficiency signaling the onset of the plateau phase which was 10,000 FU or higher.

#### Implications for quantification

A standard curve constructed from a higher threshold is moved higher on the $C_T$ vs. $\log (X_0)$ plot as a result of threshold crossings occurring later, but the slope and efficiency estimated from the slope are not changed provided the profile shapes are the same to the threshold level. The caution for threshold standard curve quantification, that initial efficiency must be the same for all samples, both calibration and test, is included in the requirement that the shapes of all profiles be the same which can be alternately stated as requiring that the initial efficiency and fall off in efficiency that occurs as amplicon amount increases must be the same *to threshold level* for all samples. Profiles developed under the same conditions will typically conform to the new requirement some cycles beyond the end of the exponential phase but thresholds must still be set at levels where efficiency is not too low, e.g., $> 50\%$, and before differences in profiles owing to sample effects, amplification of a second target, etc. develop.

Verifying that slopes of standard curves (or efficiencies derived from the slopes) constructed from thresholds at different levels are the same validates that the calibration data, up to the highest threshold tested, meets the requirements of the method. Verifying that the predicted target amount of a test sample is consistent when estimated from two or more calibration lines constructed at threshold settings where calibration profiles are shape-congruent validates that a test sample data meets the requirements of the method.

#### Application to methods using a standard curve not based on $C_T$

  Requiring profiles to have the same shape would also apply to methods that instead of a $C_T$ use the cycle position of a different profile attribute, e.g., a derivative maximum [3]. As an aside we note that because an exponential and all its derivatives are increasing functions, a derivative maximum is always beyond the exponential phase.

## Limitations

The revised model of the threshold standard curve removes the requirement that threshold crossings occur within the exponential phase of a run, provides a way to estimate where the exponential phase ends and enables new ways to check validity of both calibration and test sample data, but does not change how a standard curve is constructed or the calculation of unknown target amount in a sample. The limitations of the threshold method are not affected by the results of this study.

## Additional file

**Additional file 1.** Data extracted from Figure 2A of [2].
